# Predictors of stress among dentists during the COVID-19 epidemic

**DOI:** 10.1038/s41598-022-11519-8

**Published:** 2022-05-12

**Authors:** Dorota Wójcik, Jan Kutnik, Leszek Szalewski, Janusz Borowicz

**Affiliations:** 1 Department of Dental Prosthetics, Medical University of Lublin, Lublin, Poland; 2 Department of Personality Psychology, The John Paul II Catholic University of Lublin, Lublin, Poland; 3 Digital Dentistry Lab Department of Dental and Maxillofacial Radiodiagnostics, Medical University of Lublin, Lublin, Poland

**Keywords:** Dentistry, Public health, Psychology

## Abstract

The aim of the study was to assess the relationship between personality traits, self-esteem and life satisfaction, and also the stress level, among dentists during the COVID-19 outbreak. In order to confirm our hypotheses, 310 active dentists aged 25–64 years who were practising during the first wave of COVID-19 in Poland were examined. The IPIP-BFM-20 self-report questionnaire was used to measure personality traits, the popular Rosenberg Self-Esteem Scale was used to assess self-esteem, the five-item Satisfaction with Life Scale was used to measure life satisfaction and the four-item version of the Perceived Stress Scale was used to measure stress. The dependent variable—stress—correlates negatively with traits in relation to the dimensions of extraversion (*r* = −0.17, *p* < 0.01), emotional stability (*r* = −0.35, *p* < 0.01) and intellect (*r* = −0.16, *p* < 0.01); it also has a negative correlation with self-esteem (*r* = −0.37, *p* < 0.01) and life satisfaction (*r* = −0.35, *p* < 0.01). Among the analysed variables, the highest correlations are observed for the following: self-esteem and emotional stability (*r* = 0.51, *p* < 0.01); self-esteem and life satisfaction (*r* = 0.49, *p* < 0.01); extraversion and intellect (*r* = 0.47, *p* < 0.01). Considering the measures of mediation relevance and pathway relevance, it can be assumed that both self-esteem and life satisfaction have a mediating function in the relationship between agreeableness, emotional stability and stress: the relationship between emotional stability and stress is negative whereas that between agreeableness and stress is positive.

## Introduction

On 11 March 2020, the World Health Organization (WHO) declared coronavirus disease 2019 (COVID-19) caused by severe acute respiratory syndrome coronavirus 2 (SARS-CoV-2) to be a pandemic. Since then, most countries affected have implemented many restrictions for their citizens. The ability to leave home and to work in many industries has been restricted and teaching has moved to a remote form. On 16 March, the American Dental Association (ADA) advised dentists to curtail dental procedures for 3 weeks and perform only the minimum necessary in emergency cases. On 25 March, similar steps were taken by the National Health Service in the United Kingdom^1^. In Poland, dental surgeries, as with health centres, were not forced to close by the government and the final decision on whether to provide dental care to patients was left to dentists. However, in the face of the new threat, many dental practice owners decided to close their practices in order to adapt procedures and equipment to the new situation. SARS-CoV-2 is present in the saliva of affected patients, including those who are asymptomatic, so its spread is mainly related to respiratory droplets and aerosols. Given the nature of dental procedures, the risk of infection in the practice is high among patients, dentists and staff. The situation, similar to the SARS-CoV outbreak in 2003^2^, has demanded increased emphasis on the use of personal protective equipment by dental professionals, such as disposable coveralls, ffp3 masks, visors, caps and shoe protectors^3^.

People at risk of disease suffer from increasing psychological stress, which is exacerbated by the lockdown conditions. The psychological impact of similar conditions on people has already been reported during previous epidemics (Sars-CoV in 2003 and influenza type A subtype H1N1 in 2009)^2,4^. In China, an increase in anxiety and psychological distress was observed in the general population at the start of the epidemic^5^. The impact of the pandemic on increased levels of stress and psychiatric symptoms is evident worldwide^6–8^. The risk of anxiety, fear and psychological distress is not limited to the general population; healthcare workers in close contact with sick and infected patients have reported various symptoms of anxiety^9–11^. It is healthcare workers who are most susceptible to increased levels of stress and anxiety because they have to adapt to new workplace conditions in a short period of time and come into contact with diseased people in the course of their work duties^12^. The media and Internet portals further increase the levels of stress and anxiety by publishing new data daily on the incidence and number of deaths due to COVID-19. Such conditions foster a deterioration of mood and self-confidence. The temporary closure of workplaces and the uncertainty of workers about when to return to work have had an impact on the economic situation, which may also be related to stress and anxiety levels. Job cuts have not spared dentistry either, causing a large increase in stress levels among dentists concerned about their material status and future in the labour market^13^. Many doctors have had to return to work, specifically due to economic reasons, despite strong health concerns^13^.

In March and April, dentists in Poland were either still not working or had already returned to work, depending on the particular guidelines of the authorities and scientific societies. This brought about many changes in the equipment used, as well as in the approach to the dental team's collaboration and interaction with patients. Such changes can affect the psychosocial state of dentists in different ways. Our aim is to assess the relationship between psychological stress and dentists' concerns during the initial phase of the COVID-19 outbreak. We sought factors to explain and understand the determinants of stress levels, first by searching for potential predictors of stress among personality traits and then by considering variables that might play a mediating role between traits and perceived stress, assuming these to be self-evaluative factors such as self-esteem and life satisfaction.

At the beginning of the study, three hypotheses were formulated:

1. Personality traits are predictors of the perceived stress of dentists in the face of the COVID-19 epidemic threat. Emotional stability should be a trait of particular relevance in this case because it refers to experiencing emotional stability and the ability to cope with negative emotions, as well as how to react in crisis situations.
2. Self-esteem is negatively correlated with the level of perceived stress of dentists during the COVID-19 outbreak.
3. Life satisfaction is negatively correlated with the level of perceived stress of dentists during the COVID-19 epidemic.

The main aim of the study was to evaluate the phenomenon of stress among dental professionals during the initial phase of the COVID-19 epidemic in Poland. The assessment of risk factors for perceived stress was combined with the assessment of factors related to personality traits, life satisfaction and self-esteem of the respondents. An analysis of the relationship between risk factors and the perceived stress of dentists during the crisis related to the onset of the COVID-19 epidemic could help to identify a group that is particularly vulnerable to stress or to identify factors that reduce the perception of stress among dentists.

## Methods

### Participants and procedure

In order to confirm the hypotheses, 310 active dentists aged 25–64 years (*M* = 38.26; *SD* = 10.18) who were practising during the first wave of COVID-19 in Poland were examined. The survey was nationwide, thus obtaining a proportional distribution in the sample according to the criterion of administrative division (see Table 1). Because the survey was carried out during the first weeks of the pandemic, we adopted a convenience sampling strategy. At the same time, we controlled the survey so that the respondents represented all districts of the country. Among the respondents 86% were female (*N* = 265), resulting in an overrepresentation of women. Nevertheless, it is worth noting that in Poland this is a feminized profession and the number of male dental practitioners does not exceed 30% (Supreme Medical Chamber, n.d.). Therefore, it can be assumed that this group of respondents reflects the structure of the population of dentists in Poland. According to data from the Supreme Medical Chamber (1 March 2021), there are 48,668 active dentists in Poland: 9780 men (21%) and 38,888 women (79%). According to Eurostat data, which follow the Polish Central Statistical Office data, there are 12,200 actively employed dentists.

**Table 1:** Demographic categories: gender, place of residence, professional practice.

Demographic categories		Frequency	%
Gender	Female	265	86.5
	Male	45	14.5
Place of residence—voivodeship (region)	Lower Silesian	28	9
	Kuyavian-Pomeranian	9	2.9
	Lubusz	2	0.6
	Łódź	13	4.2
	Lublin	44	14.2
	Lesser Poland	29	9.4
	Masovian	47	15.2
	Opole	5	1.6
	Podlaskie	11	3.5
	Subcarpathian	22	7.1
	Pomeranian	13	4.2
	Holy Cross	12	3.9
	Silesian	32	10.3
	Warmian-Masurian	6	1.9
	Greater Poland	24	7.7
	West Pomeranian	13	4.2
Place of residence	Urban	270	87.1
	Rural	40	12.9
Professional practice	0–5 years	93	30
	6–10 years	61	19.7
	11–20 years	73	23.5
	21 years and more	83	26.8

Respondents completed an online survey containing a series of questionnaires. The study was performed using an online questionnaire that included questions regarding place of residence and range of professional practice. Ethical approval (Number KE-0254/90/2020) was obtained from the Research Bioethics Committee at the Medical University in Lublin. The purpose of the study and the ethical requirements for research with human participants were described (participation was anonymous and voluntary). The consent sheet appeared under the information sheet; demographic questions and all scales were presented on the remaining pages. Our questionnaire was shared online via Google with various communities and was published along with others at: Polish Dental Association official Facebook profile; Polish Dental Association local branch in Lublin Facebook profile; Tomorrow Tooth Facebook group; Style Italiano official groups; "Będąc Młodym Dentystą" Facebook site for young dentists and some other groups for dentists; and infodent24.pl, an all-Poland site for dentists. The respondents were informed about the purpose of the study and informed consent was obtained from all subjects. Research was conducted in accordance with the Declaration of Helsinki.

Data collection was from 22 February to 30 April 2020. We provided power analysis for the basic model, which included seven predictors of stress (the Big Five, self-esteem and life satisfaction) and two control variables (gender and age). The G*Power program was used to perform the calculations. For the nine predictors, by considering the size of expected effects (f^2^ = 0.11), at the significance level of α = 0.05 and performing a priori analysis we estimated a sample size of 204 for the statistical power of 0.95.

### Measures

The study used short questionnaire tools. The Polish adaptation of the IPIP-BFM-20 self-report questionnaire was used to measure the Big Five personality traits^14,15^. The questionnaire consists of 20 items and is used to examine five basic personality traits: extraversion, agreeableness, conscientiousness, emotional stability and intellect. Respondents give their answers on a five-point scale from 1 (*completely does not describe me*) to 5 (*completely describes me*). Cronbach's alpha coefficient values in this study were: extraversion, 0.86; agreeableness, 0.7; conscientiousness, 0.77; emotional stability, 0.76; intellect, 0.62.

The Polish version of the popular Rosenberg Self-Esteem Scale (RSES) was used to measure self-esteem^16,17^. By filling out this questionnaire, the respondents address 10 self-descriptive statements about self-perception on a four-point scale (1, *strongly agree*; 4, *strongly disagree*). The questionnaire allows the level of general self-esteem of the person to be examined. Cronbach’s alpha coefficient was 0.87.

The level of life satisfaction was measured using the Polish version of the five-item Satisfaction with Life Scale^18,19^. This questionnaire aims to assess the level of current life satisfaction: it measures satisfaction with life at a given moment. The items are scored on a seven-point scale from 1 (*strongly disagree*) to 7 (*strongly agree*). Cronbach’s alpha coefficient was 0.89.

In order not to make the study too extensive, the popular four-item version of the Perceived Stress Scale was used to measure stress^20^. The Polish version is also popular due to its good psychometric properties^21^. On a five-point scale, the respondent assesses how often (from "never" to "very often") certain feelings and thoughts related to stress have occurred to him/her during the past month. The questionnaire consists of four statements. Cronbach's alpha in this study was 0.74.

Pearson correlation analysis was performed to determine the relationship between the variables under study. Then, in order to assess the structural relationships between significantly correlated variables, structural equation modelling (SEM) was performed. The following software was used to perform the analyses: IBM SPSS Statistics 27 and IBM AMOS 27. Regarding the SEM procedure, the model fit test was applied according to the values of the basic fit coefficients: chi-square (χ^2^), comparative fit index (CFI), standardized root mean square residual (SRMR) and root mean square error of approximation (RMSEA). The analysis was done in a stepwise fashion and we removed irrelevant parameters and pathways from the model, according to the modification indices, to obtain a better fit. Modifications were made only if justified by theoretical knowledge of the Big Five trait relationships and the psychology of quality of life and stress.

## Results

### General characteristics of the results obtained

No missing data were identified in the sample. The survey instrument used allowed only complete responses to be recorded. As a first step, we examined the importance of professional practice. Comparisons between different groups distinguished by length of practice showed no statistically significant differences regarding the variables analysed. In further order, the correlational relationships between the analysed variables were determined. These are presented in Table 2, together with basic descriptive statistics.

**Table 2:** Mean, standard deviation and Pearson's *r* correlations between the Big Five, self-esteem, life satisfaction and stress levels.

	Variable	*M*	*SD*	1	2	3	4	5	6	7	8
1	Age	38.26	10.18	–							
2	Extroversion	12.10	3.9	−0.01	–						
3	Agreeableness	15.60	2.6	0.06	0.29**	–					
4	Conscientiousness	14.38	3.43	0.2**	0.01	0.19**	–				
5	Emotional stability	10.75	3.16	0.1	0.31**	0.09	−0.01	–			
6	Intellect	15.00	2.71	−0.03	0.47**	0.22**	0.12*	0.2**	–		
7	Self-esteem	30.05	4.72	0.03	0.35**	0.15**	0.09	0.51**	0.28**	–	
8	Life satisfaction	21.87	6.09	−0.02	0.2**	0.12*	0.08	0.31**	0.13*	0.49**	–
9	Stress	8.26	2.83	0.05	−0.17**	0.08	0.02	−0.35**	−0.16**	−0.37**	−0.35**

* *p* < 0.05; ** *p* < 0.01.

Of all the analysed traits, only conscientiousness correlates at a low level with age. The dependent variable—stress—correlates negatively with traits in relation to the dimensions of extraversion, emotional stability and intellect; it also has a negative correlation with self-esteem and life satisfaction. Among the analysed variables, the highest correlations are observed for the following: self-esteem and emotional stability; self-esteem and life satisfaction; extraversion and intellect.

### Verification of hypotheses using path analysis

The initial model that included all the Big Five characteristics was found to fit the data well. The fit coefficients had the following values: χ^2^/df = 2.635; *p* < 0.005; CFI = 0.965, RMSEA = 0.073 [CI = 0.013–0.107]; SRMR = 0.051. Nevertheless, after path analysis, it appeared that there was no main effect (significant relationship) between the dependent variable—stress—and the extraversion, intellect and conscientiousness traits. Age and gender were included in the analysis as control variables, but this did not improve the fit of the model.

After including the path coefficients, the model was tested again and an even better fit to the data was obtained, meeting more stringent fit criteria. A model considering only emotional stability and agreeableness was found to fit the data well: χ^2^/df = 1.627; *p* = 0.197; CFI = 0.995, RMSEA = 0.045 [CI = 0.001–0.13]; and SRMR = 0.033. The model explains 23% of the variance in stress.

**Figure 1:**
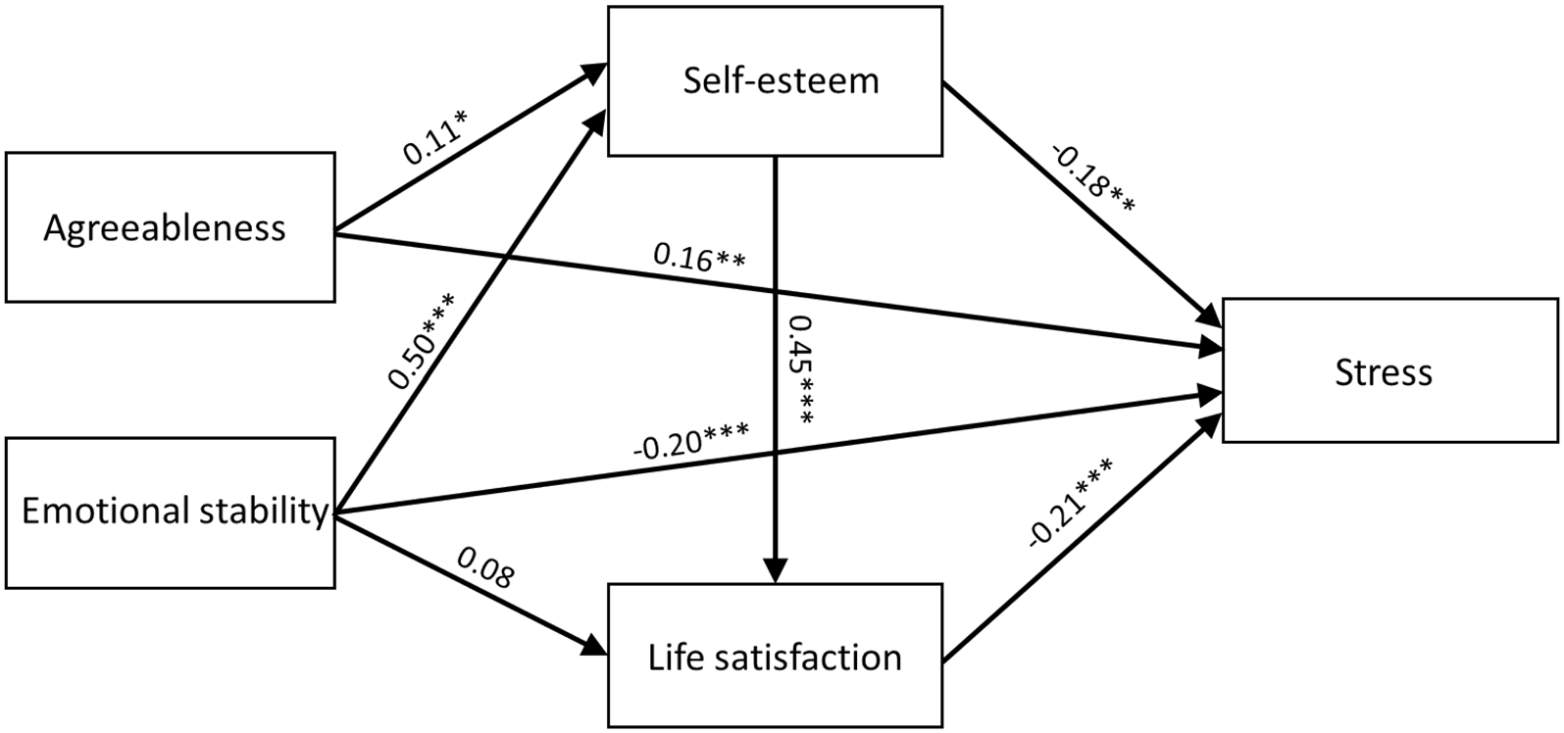
Fitted model of the relationship between agreeableness, emotional stability, self-esteem, life satisfaction and stress (standardized coefficients). * *p* < 0.05; ** *p* < 0.01; *** *p* < 0.00.

In this model, all pathways turned out to be statistically significant, except for the pathway between emotional stability and life satisfaction. Both pathways indicating a main effect between measured traits and stress turned out to be significant. The mediating effect of self-esteem and life satisfaction on the relationship between traits and stress was then calculated. A bootstrapping procedure was used to estimate the significance of the mediation effect. Mediation pathways which indicated a significant mediation effect are presented in Table 3) (Fig. 1).

**Table 3:** Bootstrapping to determine standardized indirect effects.

Model pathways	Estimates	95% CI	
		Lower	Upper
Agreeableness → self-esteem → stress	− 0.02	− 0.06	− 0.01
Agreeableness → self-esteem → life satisfaction → stress	− 0.01	− 0.03	− 0.01
Emotional stability → self-esteem → stress	− 0.08	− 0.15	− 0.03
Emotional stability → self-esteem → life satisfaction → stress	− 0.04	− 0.08	− 0.02
Emotional stability → life satisfaction → stress	−0.02	−0.05	0.01

Taking into account measures of mediation relevance and pathway relevance, it can be assumed that both self-esteem and life satisfaction have a mediating function in the relationship between agreeableness, emotional stability and stress: the relationship between emotional stability and stress is negative whereas that between agreeableness and stress is positive. Furthermore, life satisfaction should be seen as a mediator that interacts not directly with traits but indirectly through self-esteem.

## Discussion

Our study is the first to address personality traits, perceived anxiety and life satisfaction among dentists during the initial phase of the COVID-19 outbreak in Poland. The main aim of the study was to understand the importance of personality traits: which of them indicate vulnerability to stress or may be a protective factor against perceived stress during the initial phase of the COVID-19 epidemic. We also investigated the predictive significance of personality traits in relation to self-esteem and their relationship with the perceived life satisfaction and stress of dentists.

Many years of research have helped to develop a consensus on structuring and defining the structure and essence of core personality traits using the Big Five (extraversion, agreeableness, conscientiousness, emotional stability and intellect), which represent the most important core personality traits. Conscientiousness, agreeableness and openness are positively related to subjective well-being. Some studies point to the role of conscientiousness, agreeableness and intellect as predictors of perceived stress^22–25^.

Our results show that the dependent variable—stress—correlates negatively with personality traits in relation to the dimensions of extraversion, emotional stability and intellect. Similarly, the analysis by Kocjan, Kavčiči and Avsec indicates that extraversion has a direct impact on people's subjective well-being during the COVID-19 outbreak^26^. The authors' results indicate that only extraversion has a direct effect on subjective well-being, which may be due to temperamental susceptibility and stronger positive responses to potential rewards, as well as more frequent experience of positive emotions that contribute to feelings of well-being^26^. Extroverted/highly extroverted people who tend to be sociable, open, contactable may have experienced social distance rules as more stressful compared to more introverted people. Their lives (daily schedule) changed more than those of introverted people who were already able to stay in homes more often because of their disposition. In contrast, highly open people who gained more information and better understood the consequences of the pandemic were also likely to experience higher levels of stress^26^. Our study showed that the dependent variable—stress—correlates negatively with the extraversion trait.

Krok, Zarzycka and Telka demonstrated that belief in one's own efficacy was associated with experiencing less stress, which was correlated with lower life satisfaction^27^. The lower the level of perceived stress, the stronger the feeling of meaning-making and the greater the satisfaction with life and the feeling of positive emotions. On the other hand, Krok and Zarzycka investigated whether there is a relationship between risk perception of COVID-19, meaning-based resources and psychological well-being and the mediating role of coping styles among healthcare personnel in Poland^28^. COVID-19 risk perception was negatively associated with psychological well-being in healthcare personnel, whereas meaning-making-based resources have a positive correlation with well-being. In turn, our results also indicate a positive correlation between life satisfaction and self-esteem among dentists during the first months of the COVID-19 epidemic in Poland^27^. Existing studies conducted during the course of epidemics of other diseases have shown that the perceived risk of infection had a negative impact on well-being during the SARS-CoV epidemic^29^. Several studies that have been conducted in China indicate that perceived threat during the COVID-19 outbreak was associated with adverse emotional reactions—an increase in feeling negative emotions^5^.

There are many studies that treat traits as predictors of other personality characteristics and situational variables, such as perceived stress or anxiety. Nikčević et al. used path analysis and found that extraversion, agreeableness and conscientiousness were negatively correlated with three mediators—health anxiety, COVID-19 anxiety and COVID-19 anxiety syndrome—which in turn were positively correlated with the presence of generalized anxiety disorder and depressive symptoms with COVID-19 anxiety^30^. According to the Big Five classification, health anxiety correlates most strongly with neuroticism and conscientiousness. Agreeableness was negatively and directly related to generalized anxiety and depressive symptoms. In our study, the mediating function between traits and perceived stress is performed by self-esteem and life satisfaction. The particular importance of self-evaluative factors in death threats is highlighted by research in the paradigm of terror management theory^31^. We referred to this theory in our study by testing whether self-esteem can be considered a mediating buffer. Our results also show a significant predictive function of emotional stability and agreeableness for life satisfaction and self-esteem, which in turn mediate the relationship with dentists' feelings of stress during the COVID-19 outbreak. The mediating nature of self-esteem is the most pronounced result of our study and seems to support the conclusions of terror management theory. In the model, the predictive value of agreeableness and the importance of life satisfaction, although significant, should be evaluated as relatively lower. Similarly, Nikčević, et al. demonstrated a protective effect of agreeableness in predicting the severity of COVID-19 psychological distress^30^. It can be speculated that agreeableness during the isolation caused by the COVID-19 epidemic may have supported the coping (protective) mechanisms.

There are several limitations that should be taken into account when interpreting the results of our study. The first is the use of an online questionnaire, through which the authors could only reach a limited number of surveyed dentists. However, partnerships were made with local organizations to increase interaction and the visibility of the study. Next time, it would be advisable to consider reaching a larger group of respondents in order to obtain a larger study group. Furthermore, we could not apply a technique to prevent duplicate questionnaires due to the use of Google Forms.

## Conclusions

To our knowledge, this study is the first to examine the relationship between personality traits, self-esteem, life satisfaction and perceived anxiety among dentists during the COVID-19 outbreak. The study may help to assess predictors of perceived stress in this professional group under the working conditions of the COVID-19 outbreak, as well as contribute to further research on stress and stress reduction in dentists. Supporting/building positive self-esteem in dentists during the COVID-19 epidemic presumably should have a positive impact on their functioning and help them to overcome stress. Our results also show a significant predictive function of emotional stability and agreeableness for life satisfaction and self-esteem, which in turn mediate the relationship with dentists' feelings of stress during the COVID-19 outbreak. The mediating nature of self-esteem is the most prominent result of our study and seems to support the conclusions of terror management theory.

## Data Availability

The datasets generated during and/or analysed during the current study are available from the corresponding author upon reasonable request.
